# Central Nervous System Stimulants, but Not Antidepressants, Are Associated with Lower Physical Activity in U.S. Youth: NHANES 2017–March 2020

**DOI:** 10.3390/children13070914

**Published:** 2026-07-10

**Authors:** Bowen Ma, Hua Chen

**Affiliations:** 1 School of Natural Sciences, Rice University, Houston, TX 77005, USA; bowen.ma@rice.edu; 2 Department of Pharmaceutical Health Outcomes and Policy, College of Pharmacy, University of Houston, Houston, TX 77204, USA

**Keywords:** ADHD, depression, CNS stimulants, antidepressants, adolescents, children, propensity score matching

## Abstract

**Highlights:**

**What are the main findings?**

- Significantly fewer CNS stimulant users met CDC-recommended physical activity guidelines compared to a similar group of non-users (22.06% vs. 37.02%).
- Antidepressant use in children and adolescents was not significantly associated with differences in achieving recommended physical activity levels compared to non-users.

**What are the implications of the main findings?**

- There is a critical need to actively monitor physical activity patterns in youth prescribed CNS stimulants, as they are far from meeting daily recommendations.
- Public health strategies and clinical management should explicitly promote and integrate structured physical activity alongside pharmacotherapy for children with mental and behavioral disorders.

**Abstract:**

**Background/Objectives**: Although psychopharmacotherapy and physical activity may benefit children with mental disorders, the association of psychotropic medications with physical activity remains unclear. The study evaluated the association between exposure to antidepressants or central nervous system (CNS) stimulants and the likelihood of meeting the Centers for Disease Control and Prevention (CDC) physical activity guidelines (≥60 min of moderate-to-vigorous physical activity daily) among U.S. children. **Methods**: We used data from the 2017–March 2020 National Health and Nutrition Examination Survey (NHANES) to quantify medication use and physical activity duration. The exposure to antidepressants and CNS stimulants in children and their level of physical activity were captured via interviews with pediatric participants and their adult proxies. The proportion of children meeting the CDC-recommended physical activity level was compared between propensity-score-matched antidepressant users vs. non-users, as well as matched CNS-stimulant users and non-users, using Rao–Scott chi-square tests. **Results**: Among 3835 NHANES participants aged 2–17 years, representing approximately 59.4 million U.S. children, 0.89% used antidepressants, and 2.40% used CNS stimulants. After accounting for age, sex, race/ethnicity, body mass index (BMI), and family income, a smaller proportion of CNS stimulant users met physical activity guidelines compared with non-users (22.06% vs. 37.02%; *p* = 0.0075). Antidepressant use was not significantly associated with physical activity level (12.06% vs. 15.52%; *p* = 0.6342). **Conclusions**: These findings highlight the importance of monitoring activity patterns among youth prescribed CNS stimulants and antidepressants, particularly integrating physical activity promotion into public health strategies.

## 1. Introduction

Antidepressants and central nervous system (CNS) stimulants are among the most widely prescribed medications in children. Statistics from nationally representative surveys have shown that 1.6% of United States youth were taking antidepressants between 2011 and 2014 [1], and 3.5% of children aged 0–11 and 6.2% of those aged 12–19 were reported to be taking stimulants [2]. Although antidepressants were originally approved for the treatment of major depressive disorder, their use in children has expanded to include conditions such as obsessive–compulsive disorder (OCD), post-traumatic stress disorder (PTSD), and anxiety. Similarly, while CNS stimulants are widely prescribed primarily for the treatment of attention-deficit/hyperactivity disorder (ADHD), they are also used for treating narcolepsy, postural orthostatic tachycardia syndrome (POTS), and depression.

Other than pharmacotherapy, research suggests that regular physical activity can improve several core symptoms of depression and ADHD, particularly in children and adolescents. A 2023 meta-analysis demonstrates that structured aerobic sessions led to improvements in depressive symptoms [3]. In line with these data, the U.S. Centers for Disease Control and Prevention (CDC) reports that children who meet the 60-min-per-day activity guideline experience fewer depressive symptoms [4], and current clinical guidelines often recommend integrating physical activity into the management of pediatric depression [5]. Likewise, for children with ADHD, a meta-analysis found significant improvements in attention, hyperactivity, and impulsivity for those who were prescribed aerobic exercise [6].

However, the impact of pharmacotherapy on depression remains inconclusive. The theoretical rationale linking antidepressants to physical activity behavior involves several potential pathways. On one hand, successful treatment of depression may improve motivation, energy, sleep quality, and psychomotor function [7], thereby facilitating greater participation in physical activity [8]. On the other hand, antidepressants may cause adverse effects such as sedation, fatigue, and blunted rest-activity rhythms that could reduce physical activity [9,10]. A 2022 review suggests that antidepressant side effects like fatigue or lethargy may themselves discourage exercise in children, but most studies included in the review enrolled small samples (often fewer than 10 participants) and had follow-up periods that rarely exceeded the day of testing, with the longest lasting only 10–14 days [11]. Conversely, in adults, Jensen-Otsu and Austin found that taking antidepressants had no effect on self-reported physical activity [12].

The effect of CNS stimulants on general physical activity duration is even less studied than antidepressants. A study of children with ADHD found that they moved 16–22% less on days when they were taking stimulant medication compared with days when they were not [13]. Because CNS stimulants curb spontaneous movement and hyperactivity in youth, they may also inadvertently reduce their participation in structured physical activity. In addition, CNS stimulants are widely reported to have appetite-suppressing effects [14]. This reduction in caloric intake often results in negative energy availability, potentially leaving youth fatigued and without the metabolic reserves required to sustain participation in energy-demanding physical activities. However, analyses of stimulants’ effects on moderate-to-vigorous physical activity, as the CDC describes, have yet to be conducted. This gap highlights the need for broader, population-level analyses such as the present study.

The interplay between antidepressant or stimulant use and physical activity in the management of children with mental disorders remains significantly understudied. Uncovering how medication use interacts with activity patterns could help increase the effectiveness of treatment strategies for conditions such as depression and ADHD. Therefore, the objective of this study is to examine the association between antidepressants, CNS stimulants, and physical activity in U.S. children aged 2–17.

## 2. Materials and Methods

### 2.1. Data Source

A cross-sectional analysis was performed using data from the 2017–March 2020 National Health and Nutrition Examination Survey (NHANES) [15], a representative sample of the non-institutionalized United States civilian population. Due to the COVID-19 pandemic, the 2019–2020 NHANES was not completed and thus was combined with the 2017–2018 NHANES to create a partial, 4-year dataset. Data were collected from interviews, with the questionnaire section completed by an adult proxy for children < 16, whereas adolescents aged 16 and 17 answered for themselves.

We used data for children aged 2–17 years, including information on demographics, medication use, and physical activity.

### 2.2. Exposure

The Dietary Supplements and Prescription Medication section of the Sample Person Questionnaire collects information on the use of dietary supplements, non-prescription antacid medication use, and prescription medication use. Personal interviews, using the Computer-Assisted Personal Interviewing (CAPI) system, were conducted in the home to collect data on current medication use. Survey participants were first asked, “In the past 30 days, have you used or taken medication for which a prescription is needed?” Then, to ensure accuracy, participants were asked to show medication containers for all prescription drugs, and the drug name was recorded by the interviewer.

Antidepressant Medication Use: There were seven sub-subcategories of antidepressants within psychotherapeutic agents: selective serotonin reuptake inhibitors (SSRIs), tricyclics, monoamine oxidase inhibitors, phenylpiperazine antidepressants, tetracyclics, serotonin–norepinephrine reuptake inhibitors (SNRIs), and miscellaneous antidepressants. Each antidepressant medication was recorded and eventually classified using the Multum Lexicon [16], a three-level nested category system, and those who reported any use of the seven sub-subcategories were classified as using antidepressants.

CNS Stimulant Medication Use: For CNS stimulants, there were no sub-subcategories within the CNS stimulant subcategory for CNS agents. After classification, anyone who reported use of the CNS stimulant subcategory was classified as using CNS stimulants.

### 2.3. Outcomes

Physical activity was measured using two complementary metrics: (1) a binary indicator of whether participants met the CDC physical activity guidelines and (2) a continuous measure reflecting the number of days per week participants met the recommended activity criteria. Youth activity was captured with interviewers using the CAPI system for participants aged 2–11 and 16–17 years, while those aged 12–15 self-completed the same questions in private using Audio-CASI at the mobile examination center. The participants were asked, “During the past 7 days, on how many days were you physically active for a total of at least 60 min per day?”, explicitly defining activity as an action that “increased heart rate and made you breathe hard some of the time.” Responses ranged from 0 to 7 days. Children and adolescents were classified as meeting physical activity guidelines if they reported 7 days of ≥60 min activity, per CDC recommendations [4].

### 2.4. Covariates

As reported in the literature, the level of physical activity in children is associated with their age, gender, race [17], income [18,19], and BMI [20]. Therefore, all these variables were adjusted in the analysis of the association between medication exposure and the level of physical activity. Specifically, income was recorded through a ratio of household income to the poverty level (PIR) and categorized as low-income (PIR < 1.3), middle-income (1.3 ≤ PIR < 3.5), and high-income (PIR ≥ 3.5). BMI categories were defined using age- and sex-specific percentiles according to CDC growth charts as normal weight (BMI < 85th percentile), overweight (85th to 95th percentile), and obese (BMI > 95th percentile).

### 2.5. Statistical Analysis

All statistical analyses were conducted to account for the complex, multistage probability sampling design of NHANES. To ensure nationally representative estimates and correct variance calculations, all procedures incorporated the survey primary sampling units (PSUs), pseudo-strata, and the four-year Mobile Examination Center (MEC) examination weights (WTMECPRP), reflecting the aggregated 2017–March 2020 prepandemic cycles.

Baseline descriptive statistics for the total population were generated to characterize the cohort. Differences between medication users (CNS stimulants or antidepressants) and non-users prior to matching were assessed using Rao–Scott chi-square tests for categorical variables and survey-weighted t test for continuous variables.

To mitigate observed confounding and selection bias between medication users and non-users, we utilized a greedy nearest-neighbor propensity score matching (PSM) framework. Propensity scores were calculated using survey-weighted multivariable logistic regression models, accounting for the covariates mentioned previously, and assigned to each participant. To isolate the independent effects of each medication, we ensured mutually exclusive comparison groups by excluding antidepressant users from the pool of eligible controls prior to the CNS stimulant matching process, and vice versa.

Using the estimated propensity scores, all medication users were successfully matched to non-medication users at a 1:5 ratio. This is due to the large size of the non-user group (3709 participants), which meant there were enough candidates with comparable baseline characteristics to permit a higher ratio. Matches were then executed using a caliper width of 0.25 standard deviations of the logit of the propensity score, with an exact match enforced for sex. Following the matching procedure, the balance of covariates was reassessed by calculating standardized mean differences (SMDs) to verify the elimination of baseline differences. Post-matching comparisons were then conducted using Rao–Scott chi-square tests, survey-weighted t test, and survey-weighted logistic regression restricted to the matched domain. These analyses retained the survey design parameters and weights to ensure all calculations appropriately accounted for the complex survey design.

All statistical analyses were performed with SAS version 9.4 (SAS Institute, Cary, NC, USA). All statistical inferences were based on a significance level of *p* < 0.05.

## 3. Results

**Figure 1:**
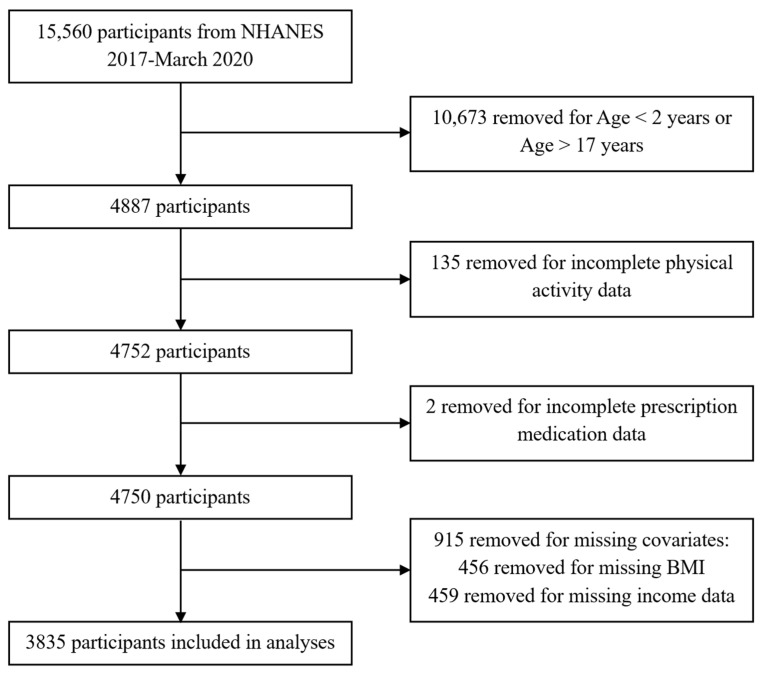
Flowchart of participant selection. A total of 3835 individuals who met the inclusion criteria were identified in the 2017–March 2020 NHANES. This cohort represents 59,404,040 children aged 2–17 in the United States.

A total of 3835 individuals who met the inclusion criteria were identified (Figure 1). This cohort represents 59,404,040 children aged 2–17 in the United States, of whom 34 (representing 543,303 children) reported taking antidepressants and 92 (representing 1,598,539 children) reported using CNS stimulants. Among the 34 individuals on antidepressants, 28 were taking SSRIs, 3 were on TCAs, 1 on SNRIs, 3 on serotonin antagonist and reuptake inhibitor (SARIs), and 2 on bupropion (both SARIs and bupropion classified as “miscellaneous antidepressants”). Additionally, 3 individuals reported using more than one type of antidepressant.

### 3.1. Factors Associated with the Receipt of Antidepressants and CNS Stimulants

Descriptive statistics before propensity score matching (PSM) presented in Table 1 indicate that, compared to the non-users, those taking antidepressants were older (*p* < 0.0001) and had a higher average BMI (*p* = 0.0002). As shown in Table 2, compared to non-users, CNS stimulant users were older (*p* < 0.0001) and were more male (*p* = 0.0021). No statistically significant differences were observed in race or household income between antidepressant or stimulant users and non-users.

### 3.2. Comparison Between Drug Users and Non-Users on Meeting Physical Activity Guidelines

Before PSM, the proportion of antidepressant users meeting the CDC guideline-recommended physical activity level was three times lower than that observed among non-users (12.06% vs. 37.57%; *p* = 0.0121). A lower rate was also observed among CNS stimulant users compared to non-users (22.06% vs. 37.57%; *p* = 0.0018). Consistently, both antidepressant users (3.83 vs. 4.81 days/week; *p* = 0.0154) and CNS stimulant users (3.93 vs. 4.81 days/week; *p* = 0.0019) reported fewer mean days per week of achieving ≥60 min of physical activity than non-users before matching.

**Figure 2:**
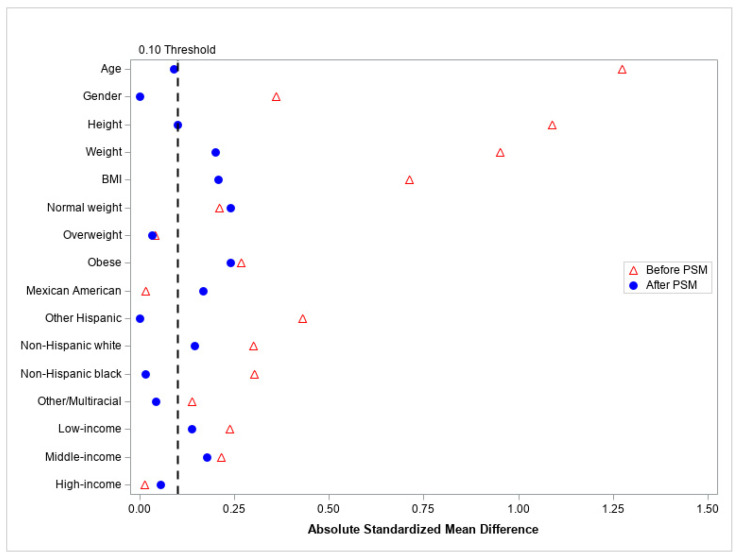
Love plot assessing covariate balance before and after propensity score matching for antidepressant users, 2017–March 2020 NHANES.

**Figure 3:**
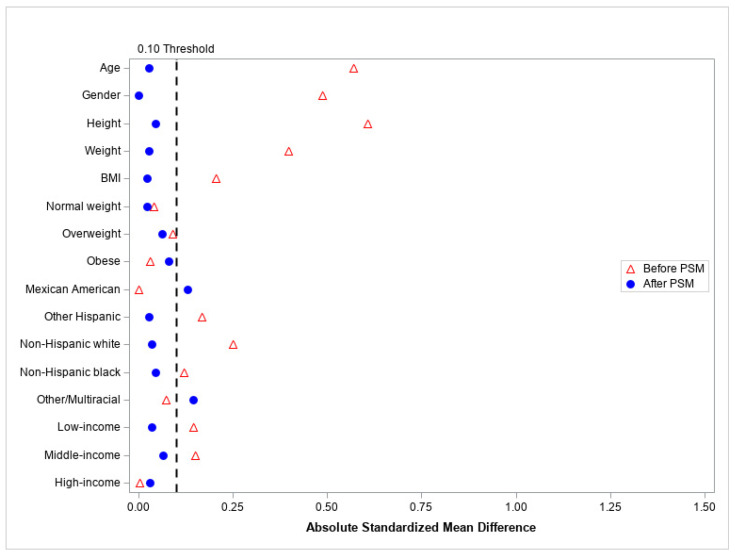
Love plot assessing covariate balance before and after propensity score matching for CNS stimulant users, 2017–March 2020 NHANES.

After PSM, all users were successfully matched: there were 170 participants matched with 34 antidepressant users, and 460 participants were matched with 92 CNS stimulant users. Due to the smaller sample size of the antidepressant group, matching substantially reduced baseline differences but left residual imbalances (SMD > 0.10) in BMI percentiles, specific racial/ethnic groups, and family income levels (Figure 2). In the CNS stimulant cohort, matching successfully balanced nearly all baseline characteristics (SMD < 0.10) (Figure 3). 

Weighted chi-square test and t test conducted between PSM matched users and non-user groups (Table 1 and Table 2) showed that there was no difference in meeting the CDC-recommended physical activity level between the antidepressant users and the matched non-users (12.06% vs. 15.52%; *p* = 0.6342). Similarly, no significant difference was observed in the mean days of physical activity per week for this group (3.83 vs. 3.67 days/week; *p* = 0.7393). Importantly, however, a significantly lower rate persists among CNS stimulant users and the matched non-users (22.06% vs. 37.02%; *p* = 0.0075), and stimulant users continued to report significantly fewer active days per week compared to their matched counterparts (3.93 vs. 4.89 days/week; *p* = 0.0021).

Sensitivity analyses were performed using survey-weighted logistic regression, with further adjustment for covariates exhibiting residual imbalance after PSM. Antidepressant use was also not significantly associated with meeting physical activity guidelines (OR = 1.159; 95% CI: 0.527–2.551; *p* = 0.7029). Conversely, youth utilizing CNS stimulants were still associated with significantly lower odds of meeting physical activity guidelines compared to matched non-users (OR = 0.482; 95% CI: 0.278–0.834; *p* = 0.0111) (Table 3), demonstrating the consistence of these findings.

## 4. Discussion

Our study is the first to examine the association between antidepressant exposure and physical activity levels in children and adolescents. Findings in our study revealed no statistically significant difference in self-reported physical activity levels between antidepressant users and non-users. This is consistent with the findings of Jensen-Otsu and Austin in an adult sample, which also reported similar levels of self-reported physical activity in antidepressant users and non-users [12], while it differs from the findings of Hirschbeck et al., which indicated that antidepressant side effects like fatigue or lethargy may discourage exercise [11].

A closer examination of our sample helps explain this null association. Among the 34 pediatric antidepressant users identified from the NHANES sample, only 6 were taking antidepressants with strong sedative effects (TCAs and SARIs) [21], whereas the majority were on SSRIs (*n* = 28). Despite sedation being reported as a side effect of SSRIs in children, its occurrence tends to be transient and can be addressed by administering the medication at the right time of day [22]. In addition, other than the adverse events that could negatively affect physical activity, the beneficial effects of antidepressants, such as improved mood, reduced anxiety, and increased energy, may affect physical activity positively. The balance of these opposing mechanisms could explain why, on average, we did not observe a statistically significant association between antidepressant exposure and physical activity.

In contrast to the findings of antidepressants, our results showed that CNS stimulant users had significantly less physical activity than non-users. Existing research on stimulants has largely examined incidental movement—such as fidgeting, restlessness, or general activity tracked by wearables—rather than intentional moderate-to-vigorous physical activity. For example, Butte et al. observed lower activity and energy expenditure in children taking stimulants, though their data were limited to a single day [13]. These findings point to a consistent reduction in spontaneous movement, but our study shifted the focus to structured, higher-intensity activity. Mechanistically, CNS stimulants increase synaptic dopamine and norepinephrine to enhance attention and reduce hyperactivity [23]. Our findings suggest that while stimulants can reduce hyperactivity, they may simultaneously lower deliberate physical activity as well.

Finally, these results have important clinical and public health implications. Although numerous studies have shown that exercise is not only beneficial for general health but also directly supports symptom reduction in those with depression or ADHD, our findings show that youth prescribed these psychotropic medications, a population that stands to benefit significantly from physical activity, are disproportionately failing to meet daily physical activity guidelines (only 22.06% of stimulant users and 12.06% of antidepressant users did so). For depression, meta-analyses in both adults and adolescents consistently demonstrate that regular physical activity has antidepressant effects and can enhance outcomes when combined with medication [24,25,26]. Similarly, reviews of exercise interventions in ADHD show improvements in attention, executive function, and core symptoms following structured aerobic activity [6,27]. This gap underscores the need to further promote physical activity alongside antidepressant and stimulant prescriptions in children and adolescents with mental and behavioral disorders.

Despite the significance, the findings of the study need to be interpreted cautiously given the inherent limitations associated with the data. The cross-sectional nature of NHANES means that no causation between medication use and physical activity can be determined. Additionally, physical activity data were self-reported and therefore may be an inaccurate measure of true physical activity levels. Future studies using objective measures of physical activity, such as accelerometers or wearable devices, could help validate and expand upon these findings. Due to the small sample size of youth antidepressant (*n* = 34) and CNS stimulant users (*n* = 92), there may be increased variability and decreased statistical power in our analyses, particularly in the antidepressant group. Finally, due to the sensitive nature of the youth mental disorders assessment [28], we were unable to adjust for underlying psychiatric conditions, an important potential confounder. Medication users differ fundamentally from non-users due to these psychiatric conditions, which themselves may directly influence physical activity levels. Failure to account for this confounding in our analysis may have biased the observed association between antidepressant and physical activity toward the null, given that children and adolescents with depression severe enough to warrant pharmacologic treatment are generally less likely to engage in physical activity than those without depression or those with milder depression not requiring medication. Likewise, the observed lower physical activity levels associated with CNS stimulants may also be affected by alternative factors such as disease severity and symptom burdens [29].

## 5. Conclusions

Given the large number of US children taking antidepressants and stimulants, it is important to understand how these medications relate to key health indicators such as physical activity. Our findings show that those taking antidepressants had no significant difference in physical activity compared to non-users, while CNS stimulant users exhibited lower physical activity levels compared to non-users. Future research is needed to clarify the mechanisms and causal pathways by which stimulants may influence physical activity and also whether physical activity can be prescribed alongside medication to treat these mental disorders.

## Figures and Tables

**Table 1:** Characteristics of antidepressant users and non-users in the 2017–March 2020 NHANES.

Variable	Before PSM				After PSM		
	Antidepressant	Non-Users	SMD	*p* Value ^1^	Non-Users	SMD	*p* Value ^1^
	(*n* = 34)	(*n* = 3709)			(*n* = 170)		
**Age, years, mean (SE)**	13.92 (0.47)	9.60 (0.13)	1.2723	<0.0001	13.43 (0.35)	0.0909	0.3215
**Male, *n* (%)**	11 (24.57%)	1847 (49.51%)	0.3603	0.0812	55 (22.84%)	0.0000	0.8739
**Height, cm, mean (SE)**	159.90 (2.23)	138.37 (0.65)	1.0874	<0.0001	156.34 (1.78)	0.1006	0.2268
**Weight, kg, mean (SE)**	63.42 (3.24)	42.42 (0.69)	0.9500	<0.0001	57.45 (2.52)	0.1993	0.1451
**BMI, kg/m^2^, mean (SE)**	24.44 (1.01)	20.18 (0.16)	0.7104	0.0002	22.95 (0.71)	0.2073	0.2118
**BMI, *n* (%)**				0.2875			0.3458
Normal (<85th percentile)	18 (49.47%)	2346 (64.26%)	0.2101		110 (64.71%)	0.2398	
Overweight (85th–95th percentile)	5 (15.69%)	601 (16.71%)	0.0415		23 (15.71%)	0.0326	
Obese (>95th percentile)	11 (34.84%)	762 (19.04%)	0.2678		37 (19.58%)	0.2402	
**Ethnicity, *n* (%)**				0.4144			0.7126
Mexican American	5 (14.32%)	526 (14.64%)	0.0149		15 (9.18%)	0.1673	
Other Hispanic	0 (0%)	314 (8.70%)	0.4300		0 (0%)	0.0000	
Non-Hispanic white	16 (63.64%)	1207 (52.20%)	0.2999		92 (70.83%)	0.1458	
Non-Hispanic black	5 (5.84%)	997 (13.35%)	0.3034		26 (7.23%)	0.0147	
Other/Multiracial	8 (16.20%)	665 (11.11%)	0.1385		37 (12.76%)	0.0436	
**PIR, *n* (%)**				0.9933			0.9684
Low-income (<1.3)	10 (30.03%)	1508 (30.11%)	0.2374		61 (32.45%)	0.1366	
Middle-income (1.3 ≤ PIR < 3.5)	16 (37.59%)	1350 (36.48%)	0.2158		65 (37.62%)	0.1786	
High-income (≥3.5)	8 (32.38%)	851 (33.41%)	0.0139		44 (29.93%)	0.0558	
**Meets PA, *n* (%)**	5 (12.06%)	1527 (37.57%)		0.0121	32 (15.52%)		0.6342
**PA, days/week, mean (SE)**	3.83 (0.39)	4.81 (0.08)		0.0154	3.67 (0.23)		0.7393

^1^ *p*-values derived from survey-weighted linear regression for continuous variables or Rao–Scott chi-square tests for categorical variables. Note: *n*, unweighted number of participants in NHANES; %, weighted percentage; PA, physical activity; PIR, poverty–income ratio; PSM, propensity score matching; SE, weighted standard error; SMD, standardized mean difference.

**Table 2:** Characteristics of CNS stimulant users and non-users in the 2017–March 2020 NHANES.

Variable	Before PSM				After PSM		
	CNS Stimulants	Non-Users	SMD	*p* Value ^1^	Non-Users	SMD	*p* Value ^1^
	(*n* = 92)	(*n* = 3709)			(*n* = 460)		
**Age, years, mean (SE)**	12.20 (0.50)	9.60 (0.13)	0.5693	<0.0001	11.78 (0.26)	0.0279	0.5049
**Male, *n* (%)**	67 (74.72%)	1847 (49.51%)	0.4866	0.0021	335 (71.97%)	0.0000	0.669
**Height, cm, mean (SE)**	153.32 (3.12)	138.37 (0.65)	0.6056	<0.0001	151.05 (1.38)	0.0460	0.5517
**Weight, kg, mean (SE)**	51.91 (3.58)	42.42 (0.69)	0.3982	<0.0001	51.28 (1.31)	0.0287	0.8771
**BMI, kg/m^2^, mean (SE)**	20.87 (0.82)	20.18 (0.16)	0.2052	0.4028	21.22 (0.28)	0.0241	0.7001
**BMI, *n* (%)**				0.6007			0.2645
Normal (<85th percentile)	60 (68.15%)	2346 (64.26%)	0.0410		295 (64.57%)	0.0227	
Overweight (85th–95th percentile)	12 (17.90%)	601 (16.71%)	0.0895		50 (12.46%)	0.0616	
Obese (>95th percentile)	20 (13.95%)	762 (19.04%)	0.0292		115 (22.98%)	0.0796	
**Ethnicity, *n* (%)**				0.4634			0.9312
Mexican American	13 (12.98%)	526 (14.64%)	0.0015		44 (10.14%)	0.1310	
Other Hispanic	4 (2.55%)	314 (8.70%)	0.1684		17 (2.74%)	0.0267	
Non-Hispanic white	41 (63.26%)	1207 (52.20%)	0.2489		213 (69.17%)	0.0360	
Non-Hispanic black	20 (9.68%)	997 (13.35%)	0.1201		91 (7.86%)	0.0457	
Other/Multiracial	14 (11.53%)	665 (11.11%)	0.0730		95 (10.09%)	0.1463	
**PIR, *n* (%)**				0.8672			0.9752
Low-income (<1.3)	44 (29.72%)	1508 (30.11%)	0.1447		212 (30.90%)	0.0351	
Middle-income (1.3 ≤ PIR < 3.5)	27 (32.85%)	1350 (36.48%)	0.1501		149 (33.55%)	0.0648	
High-income (≥3.5)	21 (37.42%)	851 (33.41%)	0.0028		99 (35.55%)	0.0311	
**Meets PA, *n* (%)**	28 (22.06%)	1527 (37.57%)		0.0018	168 (37.02%)		0.0075
**PA, days/week, mean (SE)**	3.93 (0.26)	4.81 (0.08)		0.0019	4.89 (0.13)		0.0021

^1^ *p*-values derived from survey-weighted linear regression for continuous variables or Rao–Scott chi-square tests for categorical variables. Note: *n*, unweighted number of participants in NHANES; %, weighted percentage; PA, physical activity; PIR, poverty–income ratio; PSM, propensity score matching; SE, weighted standard error; SMD, standardized mean difference.

**Table 3:** Odds ratios and 95% confidence intervals (CIs) for meeting physical activity guidelines among medication users compared to non-users, before and after propensity score matching.

	Before PSM		After PSM	
Exposure	Odds of Meeting PA Guidelines (95% CI)	*p* Value ^1^	Odds of Meeting PA Guidelines (95% CI)	*p* Value ^1^
Antidepressants vs. non-users	0.467 (0.261, 0.835)	0.0123	1.159 (0.527, 2.551)	0.7029
CNS Stimulants vs. non-users	0.228 (0.070, 0.747)	0.0167	0.482 (0.278, 0.834)	0.0111

^1^ *p*-values derived from survey-weighted logistic regression.

## Data Availability

The datasets analyzed during the current study are publicly available from the National Health and Nutrition Examination Survey (NHANES) 2017–March 2020 Pre-Pandemic Data Files, available through the Centers for Disease Control and Prevention (CDC) at https://wwwn.cdc.gov/nchs/nhanes/continuousnhanes/default.aspx?Cycle=2017-2020, accessed on 25 November 2025.

## Abbreviations

The following abbreviations are used in this manuscript:

ADHD	Attention-deficit/hyperactivity disorder
Audio-CASI	Audio Computer-Assisted Self-Interviewing
BMI	Body mass index
CAPI	Computer-Assisted Personal Interviewing
CDC	Centers for Disease Control and Prevention
CNS	Central nervous system
COVID-19	Coronavirus disease 2019
NHANES	National Health and Nutrition Examination Survey
OCD	Obsessive–compulsive disorder
PHQ-9	Patient Health Questionnaire-9
PIR	Poverty–income ratio
POTS	Postural orthostatic tachycardia syndrome
PSM	Propensity score matching
PSUs	Primary sampling units
PTSD	Post-traumatic stress disorder
SARIs	Serotonin antagonist and reuptake inhibitors
SAS	Statistical Analysis System
SD	Standard deviation
SE	Standard error
SMD	Standardized mean difference
SNRIs	Serotonin–norepinephrine reuptake inhibitors
SSRIs	Selective serotonin reuptake inhibitors
TCAs	Tricyclic antidepressants
